# General Health of Foreign-Origin Groups and Native Population

**DOI:** 10.5539/gjhs.v6n5p55

**Published:** 2014-05-09

**Authors:** Nahid Ardian, Seyed Saeid Mazloomy Mahmoudabad, Mahdi Ardian, Masoud Karimi

**Affiliations:** 1 Social Determinants of health Research Center, Shahid Sadoughi University of Medical Sciences- Yazd, Iran; 2 Marketing Management, Information Analyst at Pune University, India

**Keywords:** general health, GHQ28, immigrants, native, Yazd

## Abstract

**Background:**

Since the mental health of marginal settlers (non-native population) may affect other citizens’ health, the present study attempts to investigate the mental health status of marginal settlers of Yazd.

**Materials and Methods:**

this study was a descriptive, cross-sectional research, in which 400 of non-native and native population have participated. To study mental health status of people, a questionnaire was used. The first section of this questionnaire was the 28-item questionnaire of GHQ and the second section dealt with demographic characteristics such as age, sex, employment status, household income, and educational level of the father of the family. The collected data was analyzed using statistical operations of Pearson correlation coefficient, T Student, univariate Anova, and non-parametric Chi Square.

**Results:**

The results revealed that the average scores of general health were 20.09±9.84 and 17.04±9.54 for native and non-native population, respectively. Among subscales of general health, the highest and lowest average scores belonged to social dysfunctions, which showed a dangerous mental health status, and depression, respectively. There was significant difference between average score of general health and educational level of the father of the family (p<.001). The temporary employment and leased household differs significantly from the average score of general health among native population.

It was indicated that sex was one of the most powerful predictors of mental health and people had more mental health when they grew older.

Anxiety was the strongest predictor of general health for both groups.

**Conclusion:**

It seems that background factors such as educational level and employment status effect general health of people more than living in marginal settlement.

## 1. Introduction

Although located in central Iran and surrounded by desert, Yazd is relatively an industrial city in Iran, and concequantly has ahigher employment rate and economic opportunity, compare to other provinces. for example in 2011, the unemployment rate in South Khorasan was 6.5% and in Ilam was 21% (the lowest and highest rates in the country), while in Yazd the rate was 9.5% which was relatively good (Iran, 2011). Achieving industrial growth and development besides the growing urbanization, Yazd is faced with growing marginal settements.

Marginal settelment is a phenomenon which is made by informal and illegal settlements made by a group of people around urban areas (Park, 1926). Marginal settlers, also called slum dwellers, are culturally poor including social and physical aspects (Kalafi, Hagh-Shenas, & Ostovar, 2002). Most studies have said that the general health of non-native (immigrant) and marginal settlers (slum dwellers) was lower and it is related to the problems in family relations, jobs, housing status, and financial problems.

In most studies the general health of non-native and mariginal settlers are lower than native population (original settlers) which is related to jobs, home ownership, and financial problems (Azizi, Holakoie Naieni, Rahimi, Amiri, & Khosravizadegan, 2006; Bengi-Arslan, Verhulst, & Crijnen, 2002; Kalafi, et al., 2002). Lower socio-economic status may lead to more anxiety among marginal settlers (katialeveoque). Factors such as language, culture, and the land affect their mental health status (Knipscheer, De Jong, Kleber, & Lamptey, 2000) therefore different generations of non-native population are different in terms of mental health (Levecque, Lodewyckx, & Vranken, 2007).

Some researchers believe that critical changes in lives of immigrants endanger their mental health (Knipscheer et al., 2000). They are looking for more educational opportunity, job, public services and human rights (Carballo, Grocutt, & Hadzihasanovic, 1996) but are faced with discrimination, social isolation, unemployment, lower social status, and generation gaps (Dastjerdi & Khazaei, 2001). Physical and mental health of marginal settlers affects other citizens’ health in all aspect, that is why it is important. Marginal settlement in Yazd is potentially triggering various problems in the city. The study hypothesis said that general health of native residents, because of better social and economic status, is better than foreign-origin groups’ mental health. A study on mental health status of people may provide valuable information for managers which is useful in planning and prevention policies. This is the basis of the present study.

## 2. Materials and Methods

This study was a cross-sectional study whose sample population was selected from non-native population in marginal settlement and native population of Yazd. the result is said to be statistically significantat the 5% level and for the test power 80% was considered adequate in order to reach significance difference in health average scores.

There were 210 people in each groups; that is, 420 samples in general. The marginal settlers of four districts of Hassan Abad, Najaf Abad, Maryam Abad, and Akramiyyeh were compared with native population in other districts of Yazd. Marginal settlers were operationally defined as non-native settlers with special economic, cultural, and social status, who have resided in Yazd for at least three months and up to 10 years. At first 50 families were selected from each district. Interviewers complete questionnaires by interviewing the parents. The control group included native population selected by cluster sampling. To do that, alleys were considered as clusters in a district. Then 40 families were selected randomly in five urban districts.

The questionnaire was completed by interviewers. The used questionnaire included two sections. The first section was on demographic characteristics including sex, age, employment status, home ownership, household income, and educational level of the father. The second section was the 28-GHQ General health questionnaire. The validity of this questionnaire was confirmed by previous in these studies (Hashemi, Khosravi, Faghihzadeh, & Etemadzadeh, 2007; Movaghar & Rahimi, n.d.; Taghavi, 2002; Yaghoubi, Nasr, & Shahmohammadi, 1995; Zadik, Gilad, & Peretz, 1997) including four subscales of somatic symptoms, anxiety, social dysfunction, and depression. It was scored using a four level Likert scale, ranged from 0 to 3. Therefore the total scores ranged from 0 to 84 and the cutoff point was 23. Scores higher than 23 indicated in danger of mental health problems. Concerning scores of the four subscales, ranging from 0 to 28, the cutoff point was 14, scores higher than which were considered as the indicators of mental disorders. The collected data was analyzed by SPSS software Ver 16, IBM corporation, Armok, NY.USA, Data analysis were done by conducting several statistical operations such as t-test, chi-square, one-way ANOVA, Pearson correlation coefficient, and linear regression.

## 3. Results

Among 395 respondents participating in our study, 191 were original settlers including 132 female (70%) and 58 males (30%); Mean of age for original settlers were 42.37±14.34 and for marginal settlers were 35.18±13.11and 204 were marginal settlers including 103 males (51%) and 101 females (49%). 25% native families had only one child and 75% had two or more children, however among marginal settlers 30% of families had only one child and 70% had two or more children.

The average score of general health among native population was 20.09 ± 9.84 and among non-native population was 17.04±9.54. Among 28-GHQ subscales, the highest average score belonged social dysfunction which was 7.25± 2.32 among native population and 7.10±1.96 among non-native population, that is higher than cutoff point of 7 and the lowest average score belonged to depression which was 2.53±3.88 among native population and 1.68±3.14 among non-native. Concerning cutoff point of 23, 46 original settlers (33%) and 38 marginal settlers (23%) had obtained average scores of more than 23 (Table 2).

**Table 1:** Discriptive statistics of non-native and native population, concerning their demographic characteristics

Demographic Characteristics		Original settlers		Marginal Settlers	
		N	%	N	%
**Educational level of the father**	Illiterate	21	11	71	34
	Primary school	54	28	69	33
	Secondary school	81	43	55	27
	Diploma	32	17	11	6
	Bachelor (BA,BM)	2	1	1	
**Household income (million Rials)**	To 3	38	22	98	48
	To 5	93	23	89	44
	8	38	22	16	8
**Employment status**	permanent	108	66	111	57
	temporary	55	34	82	43
**Home ownership**	Owner	143	75	106	53
	Tenant	48	25	96	47
**District buildings and gatherings**	library	74	39	22	11
	mosque	153	80	122	58
	Sport events/gatherings	63	33		
	Religious events/gatherings	88	46	20	10

**Table 2:** Descriptive statistics of non-native and native population

	Original settlers		Marginal Settlers		t	p

	Mean	SD	Mean	SD		
**GHQ**	20.09	9.84	17.04	9.54	2.75	.006
**SS**	5.67	3.78	4.20	3.47	3.14	.000
**AL**	5.49	4.38	4.14	4.21	3.16	.002
**SD**	7.25	2.32	7.10	1.96	.65	.510
**D**	2.53	3.88	1.68	3.14	2.37	.010

SS Somatic Symptoms; AI Anxiety/Insomnia; SD Social Dysfunction; D Severe Depression; T Total GHQ–28 score.

Table 3 shows concerning employment status, the average score of general health was significantly different between these groups (p<.001). The average score of general health among those who were contractually employed (temporary employment) was 23.51±11.92 and 17.53±10.07, respectively for original and marginal settlers. Comparing the average score of general health among participants living in leased homes indicated a significant difference (p<.001) between original and marginal settlers whose scores were 23.68±9.31 and 16.64±9.22, respectively. The average scores of general health gained by original and marginal settlers, whose household incomes were lower than 3 million Rials, were significantly different. The average scores of general health gained by original and marginal families whose fathers had primary education were also significantly different.

**Table 3:** Descriptive statistics of general health and its subscales scores, concerning demographic characteristics

	Original settlers		Marginal Settlers		t	p

	Mean	SD	Mean	SD		
**Employment Status**						
**Permanent Employment**	18.35	8.52	16.32	9.51	1.47	.140
**Temporary Employment**	23.51	11.92	17.53	10.07	2.73	.007

**Home ownership**						
**owner**	19.04	9.79	17.34	10.09	1.87	.240
**tenant**	23.68	9.31	16.64	9.22	3.61	.000

**Equivalent income (million Rials)**						
**To 3**	22.16	11.45	15.85	9.74	2.86	.005
**To 5**	20.08	8.58	18.52	9.12	1.04	.290
**More than 8**	16.03	6.67	17.20	11.40	-.42	.670
**Educational level**						
**illiterate**	18.50	13.44	17.54	8.47	.36	.710
**Primary education (5years)**	21.78	9.53	15.15	8.15	3.54	.001
**Secondary education (9 years)**	20.14	9.38	19.32	11.79	.40	.680
**Diploma (12 years)**	18.80	9.77	15.60	10.13	.86	.390

The Pearson correlation coefficient matrix revealed that all subscales of GHQ were significantly different from each other and from total GHQ score (P<.005) (Table4).

**Table 4:** The Pearson correlation coefficient matrix of GHQ subscales

	GHQ		SS		AL		SD		D	

	Original	Marginal	Original	Marginal	Original	Marginal	Original	Marginal	Original	Marginal
GHQ	1	1								
SS	**.752	**.859	1	1					
AL	**.875	**.876	**.714	**.646	1	1				
SD	**.618	**.651	**.479	**.238	**.431	**.360	1	1		
D	**.705	**.717	**.404	**.380	**.562	**.533	**.398	**.411	**1**	**1**

SS Somatic Symptoms; AI Anxiety/Insomnia; SD Social Dysfunction; D Severe Depression; T Total GHQ–28 score.

** P < .0001.

Conducting Spearman correlation test, it was found that the correlation between household income of marginal settlers and their general health was significant r=.151, p<.05.

Table 5 shows that sex was one of the best predictors of mental health, that is, men had poorer mental health. Comparing different age groups, those who were older had better mental health. Based on logistic regression analysis, higher educational level led to better mental health.

**Table 5:** Estimated logistic regression confidence and odds ratios concerning demographic characteristics

	Odds	P value	95% confidence interval
Gender	1.895	.041	1.026- 3.502
Age<30	1	.017	1
30-45	.707	.041	.346 - 1.445
45-60	.400	.045	.163- .981
>60	.090	.003	.018 - .451
Home owner	.872	.696	.439- 1.732
Number of children	.873	.675	.462- 1.647
Educational level	1	.000	1
Literate	.329	.008	.144- .751
Primary education	.148	.000	.066 - .335
Secondary education	.129	.001	.038 - .435
Diploma	.000	.999	.000
Income	.410	.000	.251- .669

## 4. Discussion

The results revealed that marginal settlers were mentally healthy, compare to original settlers. It was in contrast with some studies (Azizi et al., 2006; Kalafi et al., 2002; Motaghipour et al., 2006) which concluded that non-native population was were less healthier than native population. However, this finding was in accordance with the mental health status reported in the province and with other similar studies all around the country (Halvani, Morovati Sharifabad, & Baghiani Moghadam, 2007; Lotfi, Aminian, Ghomizade, & Zarea, 2010; Nastiezaie, Vaezi, Molazahi, & Moghadam, 2008; Noorbala, Yazdi, Yasamy, & Mohammad, 2004). Most of the marginal settlers in the four investigated districts were immigrants coming from rural areas or other towns within the same province. Therefore; they were slightly different from native population in terms of accent, culture, geographical aspects, attitudes and beliefs. It is possible to consider their cultural and social properties the same as original settlers’. To explain more the average a core of general health, it can be said that according to some socialists, thinking and social lives of different classes of people are different. People of upper, middle- and lower class may have different attitudes towards various problems (Boudreau, 1999) and mental health depends on socio-economic factors, as mentioned in many studies (Knipscheer et al., 2000; Levecque, et al., 2007). In the present study native population had better economic status, compare to marginal settlers. Although definition of middle class and its components have changed or is changing, some theorists defined middle class as a part of labor class or a socio-economic classification system whose social status and welfare state is neither upper nor lower (Hezarjaribi & Safari, 2010) Sociological studies showed that middle class is usually more dissatisfied than upper and lower class. It can be said that middle class is more critical than upper and lower class (Boudreau, 1999; Hezarjaribi & Safari, 2010) The present study confirmed this idea, so that native population who seemed to have better socio-economic status compare to marginal settlers, were more dissatisfied and consequently had relatively poorer general health.

The result showed that sex as a variable had influences on individuals’ mental health. This study, contrary to some other studies indicated that the mental health among women is better than men. Considering the lifestyle, occupation and problems which men have to deal with in our the society, they are probably in danger, more than women, in terms of mental health.

Results revealed that age can be considered as a predictor of mental health. In this study, older people had better mental health. Although other studies (Evans, Fletcher, & Wormald, 2007; Noorbala, et al., 2004) reported an opposite conclusion, it seemed that Iranian men are confronting some economic and social problems which negatively affect their mental health.

Social dysfunction has been usually seen in studies on workers’ general health. Concerning demographic characteristics such as household income, educational level, and employment status, social dysfunction and depression received the highest and lowest scores respectively, which is similar to other studies (Dastjerdi & Khazaei, 2001; Halvani et al., 2007; Taghavi, 2002).

It seems that the problems mostly affect social dysfunction and then the other subscales e.g. somatic symptoms, anxiety, and depression got involved. The hierarchy of subscales of GHQ is as follows: social dysfunction, anxiety, somatic symptoms, and depression. As Goldberg (Goldberg et al., 1997) had said, to use the questionnaire it is not necessary for subscales to be similarly important. Also there is no need to have similar factor structure among samples. The depression was rarely seen in both groups which may result from the strong religious beliefs among people and types of interpersonal relations in a traditional community and different socio-economic factors mostly affect their social dysfunction. Employment status is one of the most effective factors in general health and its effect was confirmed in many studies. Concerning different jobs, employment type, shift work, working time (Hashemi et al., 2007; Movaghar & Rahimi, n.d.; Saberi et al., 2009) endanger general health’s scores among native population who were temporarily employed showed their endangered mental health. However there was no significant difference between general health’s scores and employment status of non-native population. It seems that original settlers who have better welfare state pay more attention to their employment type, therefore, whenever their employment status and consequently their economic status are in danger; their mental health is also in danger. The same explanation can accounts for home ownership. Original settlers living in leased homes were mentally less healthy, since their expectations were higher. Comparing non -native and native population in terms of household incomes and average scores of general health, there was a significant difference.

Comparing those earning less than 3 million Rials, the average scores of general health was significantly different between the groups. However there was not noticeable difference between families which earn more than 3 million Rials. It seems that mental sensitivity of native population who earn lower income is more than their counterparts among non-native population, while those who have medium or higher incomes are relatively similar in both groups. Perhaps the higher the income, the better the general health, for both groups. Educational level is an effective factor in mental health and one of the factors preventing mental disorders (Noorbala & Mohammad, 2009; Palahang, Nasr, & Shahmohammadi, 1996). The positive correlation between educational level of the fathers and the general health seemed reasonable. Results showed that as the educational level increase, the general health improve more. Among native population the lowest scores of mental health was received by illiterate fathers. Some studies concluded that there was a negative correlation between the educational level of the fathers and the average score of general health, that is, the higher the educational level, the lower the scores of general health (Bahrami, Bagherpour, Fathi Ashtiani, & Ahmadi, 2009; Halvani et al., 2007; Hashemi et al., 2007; Movaghar & Rahimi, n.d.).

However educational level had not significantly effect on general health of marginal settlers. In fact, many of them were illiterate or had primary education; therefore there was no significant correlation. It was not in accordance with several studies done before (Dastjerdi & Khazaei, 2001). It seems that when more than 90% of marginal settlers have no education or at most primary education, their general health is not related to their education and as stated in Kinship’s study social and cultural backgrounds more than marginal settlement affects their health (Knipscheer et al., 2000). Concerning the family size, the number of children in native families were more than non-native population, which may refer to the fact that immigrant families are younger than native ones.

Social support is another factor which had some effects on general health (Levecque, et al., 2007) which can decrease various stressors, and lower stress results in better mental health (Esfandiari, 2001). Different gatherings such as religious ceremonies and sport events are classified as samples of communication opportunities and social support (Norbeck & Tilden, 1983; Vaux et al., 1986). Data collected in this study had shown that there were more mosques in original settlement than marginal settlement. Mosque was the main gathering place and facility stated by original and marginal settlers. Although in marginal settlements there were fewer religious and sport gatherings, the settlers were relatively healthy. It may be explained by the fact that there were other types of social supports such as close relationships between family relatives and neighbors or other types of relations which were particular in each district.

## 5. Conclusion

The results revealed that marginal settlers were mentally healthy, compare to original settlers. It seems that background factors such as Social stratification, educational level and employment status effect general health of people more than living in marginal settlement.

**Limitation**

One of the limitations of this study was the fact that immigrants of Yazd came from rural areas or other towns around Yazd and foreign immigrants like Afghans and Arabs did not live in districts under investigations. Different results were expected if foreign immigrants were included.
